# The selection of copiotrophs may complicate biodiversity-ecosystem functioning relationships in microbial dilution-to-extinction experiments

**DOI:** 10.1186/s40793-023-00478-w

**Published:** 2023-03-17

**Authors:** Zhendu Mao, Zifan Zhao, Jun Da, Ye Tao, Huabing Li, Biying Zhao, Peng Xing, Qinglong Wu

**Affiliations:** 1 State Key Laboratory of Lake Science and Environment, Nanjing Institute of Geography and Limnology, Chinese Academy of Sciences, Nanjing, 210008 China; 2 Center for Evolution and Conservation Biology, Southern Marine Sciences and Engineering Guangdong Laboratory (Guangzhou), Guangzhou, 511458 China; 3 University of Chinese Academy of Sciences, Beijing, 100049 China; 4 College of Life Science, Anhui Normal University, Wuhu, 241002 China; 5 International Genome Center, Jiangsu University, Zhenjiang, 212013 China

**Keywords:** Dilution-to-extinction, Community assembly processes, Biodiversity-ecosystem functioning

## Abstract

The relationships between biodiversity-ecosystem functioning (BEF) for microbial communities are poorly understood despite the important roles of microbes acting in natural ecosystems. Dilution-to-extinction (DTE), a method to manipulate microbial diversity, helps to fill the knowledge gap of microbial BEF relationships and has recently become more popular with the development of high-throughput sequencing techniques. However, the pattern of community assembly processes in DTE experiments is less explored and blocks our further understanding of BEF relationships in DTE studies. Here, a microcosm study and a meta-analysis of DTE studies were carried out to explore the dominant community assembly processes and their potential effect on exploring BEF relationships. While stochastic processes were dominant at low dilution levels due to the high number of rare species, the deterministic processes became stronger at a higher dilution level because the microbial copiotrophs were selected during the regrowth phase and rare species were lost. From the view of microbial functional performances, specialized functions, commonly carried by rare species, are more likely to be impaired in DTE experiments while the broad functions seem to be less impacted due to the good performance of copiotrophs. Our study indicated that shifts in the prokaryotic community and its assembly processes induced by dilutions result in more complex BEF relationships in DTE experiments. Specialized microbial functions could be better used for defining BEF. Our findings may be helpful for future studies to design, explore, and interpret microbial BEF relationships using DTE.

**Supplementary Information:**

The online version contains supplementary material available at 10.1186/s40793-023-00478-w.

## Introduction

Microbes are key components of biodiversity and play important roles in ecosystem functioning [1, 2]. For a long time, the ecosystem functions of the microbial community were thought as highly redundant [3, 4] because the diversity of the microbial community is tremendous [5], and the functional genes are highly redundant [6]. Recently, a few studies have suggested that the loss of biodiversity in microbial communities also impairs the ecosystem functioning in different ecosystems [7–9]. In fact, the number of biodiversity-ecosystem functioning (BEF) studies in microbial communities is much fewer than the number of BEF studies in macroscopic communities, which does not match the important roles of microbes in different ecosystems [10, 11].

In recent years, the development of high-throughput sequencing has enabled the quantification of microbial diversity and thus facilitated the exploration of microbial BEF [12]. Dilution-to-extinction (DTE) has mostly been used to manipulate microbial diversity to study microbial BEF relationships in recent years [13, 14]. During DTE, the high dilution level could reduce the abundance of species and then remove rare species to obtain lower diversity [15]. DTE has become an important method to study microbial BEF relationships [10, 12, 16, 17] and provides evidences that rare species play vital roles in ecosystem functioning [15, 18, 19].

An important advancement of ecology in the last twenty years is the understanding of how stochastic processes contribute to assembling communities [20, 21]. Now, it is well recognized that stochastic and deterministic processes shape the community together, but their relative importance in community assembly may vary [21, 22]. It is also interesting to know how community assembly processes determine functional performance [22]. For example, many researchers believe that different community assembly processes will change BEF relationships [23–25]. A model study and an experimental study based on microbial communities showed that the dominance of stochastic processes would impair ecosystem functioning generating negative BEF relationships, and deterministic processes could result in positive BEF relationships [24, 26]. Thus, understanding how microbial communities are assembled during DTE experiments is very important for in-depth analysis of the microbial BEF relationships.

In ecology, there is a fundamental life strategy-based spectrum running from *r*-strategists, which achieve their instinct growth rate (*r*_max_) when resources are sufficient, to *K*-strategists, which maintain their population size near the carrying capacity (*K*) when resources are limited [27]. In microbial ecology, there is a framework similar to this spectrum. It is the copiotroph–oligotroph spectrum, where copiotrophs are thought to be fast-growing while oligotrophs are thought to grow slowly and efficiently [28]. The ribosomal RNA operon (*rrn*) copy number in the microbial genome is a candidate index for distinguishing copiotrophs and oligotrophs because of its good prediction of maximum growth rates [29–32]. In a primary succession of microbial community, copiotrophs, those with high *rrn* copy numbers, are dominant in early succession and later replaced by oligotrophs, those with low *rrn* copy numbers [33–35]. The abundance-weighted mean *rrn* copy number at the community level consequently reduced with succession of microbial community [33, 35]. Therefore, the application of *rrn* copy number fitted the understanding of copiotroph-oligotroph spectrum and could help reveal processes behind community dynamics.

There are some suggestions that a higher dilution level might result in a higher ratio of copiotrophs [10, 17], because the available nutrient level is relatively high compared to the low microbial abundance in diluted communities. However, this possibility has rarely been studied. This possibility should not be neglected as copiotrophs and oligotrophs have contrasting functional traits and performance [28], which may influence BEF relationships in DTE studies. On the one hand, the selection of copiotrophs could contribute to the dominance of deterministic processes at high dilution levels. On the other hand, the community assembly of rare species is driven mainly by stochastic processes [36, 37]; The stochastic processes could be weakened as the loss of rare species is an important process occurring at higher dilutions in DTE experiments [15]. In this study, we conducted a microcosm study using DTE and further verified our results using a meta-analysis of DTE studies. We aimed to determine how the selection of copiotrophs and loss of rare species in DTE contributed to the community assembly and how the shift in microbial community assembly processes would influence the BEF relationships.

## Methods

### A microcosm study

The original microbial communities were bacterioplankton communities from the surface of Lake Zixia (118.84424° E, 32.06042° N), Jiangsu Province, China. All microeukaryotes larger than 0.8 µm were excluded by subsequently filtering the water once through 5-µm and twice through 0.8-µm Isopore membrane filters (Millpore, Massachusetts, USA). The removed microeukaryotes include flagellates and ciliates, which feed on bacteria, and the existence of predators could cause the dominance of oligotrophs [38]. The filtered community was set as the initial community. Rest lake water was autoclaved (20 min at 121 ℃) and used as the medium for community regrowth. We prepared a 5-step dilution gradient with a dilution factor of 1:10, yielding 6 diversity levels with a sterile level as control. In brief, 200 mL of the initial community was transferred into a 3-L conical flask with a 1.8 L final of autoclaved lake water and the bottles were covered by semi-permeable membrane to prevent air contaminations. This procedure was repeated four times to get the highest dilution of 10^− 5^. After dilution, all the bottles were placed in a dark indoor environment with a room temperature of about 23℃. We shook the bottles twice a day for the oxygen supply and then randomly swapped the positions of the different bottles. We sampled each bottle for cell density every two days. After 8 days of inoculation, we collected three replicates for each dilution level. Approximately 600 ml of the collected water was filtered through 0.2-µm Isopore membrane filters (Millipore, Billerica, MA, USA) and later stored at − 20 °C until DNA extraction and subsequent sequencing. These steps including dilution and sampling were conducted in a Biological Safety Cabin to avoid microbial contamination from the air.

### Analysis of 16S rRNA gene sequencing data

The filters were sheared and microbial DNA was extracted using the FastDNA Spin Kit for Soil (Mo Bio Laboratories, Carlsbad, CA, USA) according to the manufacturer’s protocols. The primer 515 F/806R for 16S rRNA gene was selected for polymerase chain reaction (PCR) amplification. Library preparation and DNA sequencing on the Illumina MiSeq platform (Illumina, Inc., San Diego, CA, USA) were performed at Shanghai BIOZERON Biotechnology Co., Ltd. (Shanghai, China). The raw sequencing data are available in the Sequence Read Archive through the project accession PRJNA864105.

We merged the sequences and stripped the primers using USEARCH (Edgar, 2010). Clustered reads were classified into operational taxonomy units (OTUs) at a 97% similarity level using the UPARSE algorithm [39] with the option of excluding global singletons from the clustering step. The chimera was also removed during the cluster operation. The taxonomic assignment of the representative sequences of OTUs was analyzed by SINTAX algorithm [40] against the Ribosomal Database Project training set [41] with an 80% confidence score. Those OTUs failing to be classified as *Bacteria* or *Archaea* were also removed for subsequent analyses.

Finally, 699,963 reads of 16S rRNA gene fragments were obtained and could be clustered into 805 prokaryotic OTUs. The OTU abundance tables were rarefied by the lowest number of OTUs (29,915 reads) using the package ‘vegan’ [42] on R Statistical Software (v4.0.5) [43]. Then, the *rrn* copy number for each OTU was predicted and the abundance was corrected to obtain the corrected cell number following the same strategy as Wu et al. [44]. First, the *rrn* copy number for each OTU was estimated based on its taxonomy using the *rrn*DB database [45]. If the child-taxon of an OTU was identified in the *rrn*DB database, the average *rrn* copy number of this child-taxon was applied; otherwise, the average *rrn* copy number of its parent-taxon was applied. Next, the abundance in OTU table was corrected, divided by the responding *rrn* copy number of OTUs to represent the cell abundance. The mean *rrn* copy number of each community was calculated using the abundance-weighted average method.

### Diversity metrics and community attributes

Species richness (also called as observed OTU number) and the Shannon index were calculated using the package ‘vegan’ [42] on R Statistical Software (v4.0.5) [43] based on the OTU abundance table after the correction of *rrn* copy number.

The modified stochasticity (MST) was calculated to present the relative importance of stochastic processes vs. deterministic processes [46]. MST reflects the contribution of stochastic processes based on relative differences between the observed situation and the null expectation and therefore can better quantitatively measure the stochasticity in community assembly [46]. The MST index defines 0.5 as the threshold to determine whether the community assembly is more deterministic (< 0.5) or more stochastic (> 0.5). Here, the unweighted distance based on Jaccard dissimilarity was used, which gives the same weight to rare species and abundant species. The calculation of MST was achieved using the package ‘NST’ [46] on R Statistical Software (v4.0.5) [43].

As rare species are more vulnerable to DTE [15], we divided different species in each community into rare species (relative abundance < 0.1% locally) and abundant species (relative abundance > 1% locally) [47, 48]. We also calculated the diversity metric and MST of rare species and abundant species. The MST difference between rare species and abundant species was tested using paired t-test.

### The BEF relationship in microcosm study

In the microcosm study, we used Biolog EcoPlate™ assays (Biolog Inc., Hayward, CA, USA) to infer carbon utilization ability as an ecosystem function [10, 49]. EcoPlates contains 31 different organic carbon substrates, and a water control in triplicate. Once the community could utilize the carbon substrate, the color of the well turned into purple and could be detected in the optical density value using a plate reader. Every 24 h, the optical density in each well was measured at 590 nm using the SynergyTM 2 plate reader (BioTek Instruments, Inc., Winooski, VT, USA) for 5 days. We calculated the blank-corrected median absorbance of each substrate and further the average well color development (AWCD) [13]. We also selected the time points when the AWCD was closest to 0.5, as suggested [13], to calculate the functional diversity. We also used the AWCD corrected by initial cell density on the third day to represent the uptake rates.

### Collecting studies for meta-analysis

The keywords used in the Web of Science search were as follows: TS = (diversity AND dilution) AND SU = microbiology. We collected 1,566 papers on March 10, 2022 (last update time). Combined with the paper referred by Roger et al. (2016) [10] and other literature cited in these papers, we finally found 127 papers describing the functioning or/and community’s structure in DTE experiments and the number of publications each year showing an increasing trend over time (Figure S1).

Articles satisfying the following criteria were used for community analyses: (1) the community structure was measured using the 16S rRNA gene amplicon sequencing method; (2) the number of dilution levels was no less than 3; (3) the raw sequencing data of sequencing could be found in Sequences Read Achieve database or other websites and their treatment information for each sequencing file could be clearly tracked based on available information. We totally collected 26 articles with 1,529 communities totally. The final studies used and their related information can be found in Table S1. The analysis of raw sequencing followed the same flow as the microcosm study. Since different primers were used for each study, we analyzed the data from different studies separately and rarefied OTU tables to their own minimum sequencing depth. We defined experiments with different community source, different culture condition and different regrowth time as distinguished experiments. We finally gained 82 experiments.

### Effect size calculation and meta-analysis

We calculated Pearson’s coefficient of correlation between each pair of dilution levels, species richness, the Shannon index, MST, and mean *rrn* copy number. We transformed Pearson’s coefficient of correlation (*r*) into a normalized effect size using Fisher’s z transformation.

1 $${z = 0.5*{\text{ln}}\left( {\frac{{1 + r}}{{1 - r}}} \right)}$$

2 $${Var\left( z \right) = \frac{1}{{n - 3}}}$$

Here, ‘n’ represents the number of observations.

All meta-analyses were performed using the package ‘metafor’ [50]. A variance-weighted mixed-model (meta regression) was applied to estimate the mean effect size (z_++_) using restricted maximum likelihood. We evaluated the heterogeneity of effect sizes among different experiments with the Q-statistic to determine whether the models could explain a significant amount of variation. Total heterogeneity (Q_t_) could be divided into the variance explained by the moderators (Q_m_, how much the moderator explains heterogeneity among different observations) and the residual error variance (Q_e_, the residual of heterogeneity remaining to be answered). We found strong heterogeneity in the meta-analysis when different correlations were calculated (Table S2). After introducing moderators, culture habitat and the duration of regrowth explained a proportion of the heterogeneity (Table S3).

### Collected BEF relationships using DTE

We collected 68 studies using DTE to study microbial BEF relationships. We used the function categories similar to the categories given by Roger et al. [10] and discarded rest ecosystem functions failed to classified into these categories. In detail, ecosystem functions could be divided into bacterial activity, degradation of carbon substrates, invasion resistance, stability (resistance, resilience, and temporal stability), plant productivity promotion, nitrogen cycling and other elementary cycling. To further understand the BEF relationships, we studied the BEF for broad functions and specialized functions. Here, broad functions are those functions that most microorganisms could perform while the specialized functions could be only carried out by specific functional groups [9, 51]. Bacterial activity and the degradation of labile carbon were defined as broad functions. Degradation of inert and xenobiotic carbon, nitrogen cycling and other elementary cycling were defined as specialized functions.

All the plots were visualized on R Statistical Software (v4.0.5) [43] by using packages ‘ggplot2’ [52] and ‘ggpubr’ [53].

## Results

### Microcosm study

Dilution-to-extinction successfully reduced species richness (Pearson’s *r* = − 0.931, *P* < 0.001) and the Shannon index (Pearson’s *r* = − 0.898, *P* < 0.001) in the microcosm study. Rare species were reduced faster than abundant species (Figure S2), showing that rare species were more vulnerable to DTE. The relative abundances of *Betaproteobacteria* and *Gammaproteobacteria* increased with increasing dilution level (*Betaproteobacteria*: Pearson’s *r* = 0.609, *P* = 0.007; *Gammaproteobacteria*: Pearson’s *r* = 0.721, *P* < 0.001), while other abundant phyla/classes decreased with increasing dilution level (Figure S3).

**Fig. 1:**
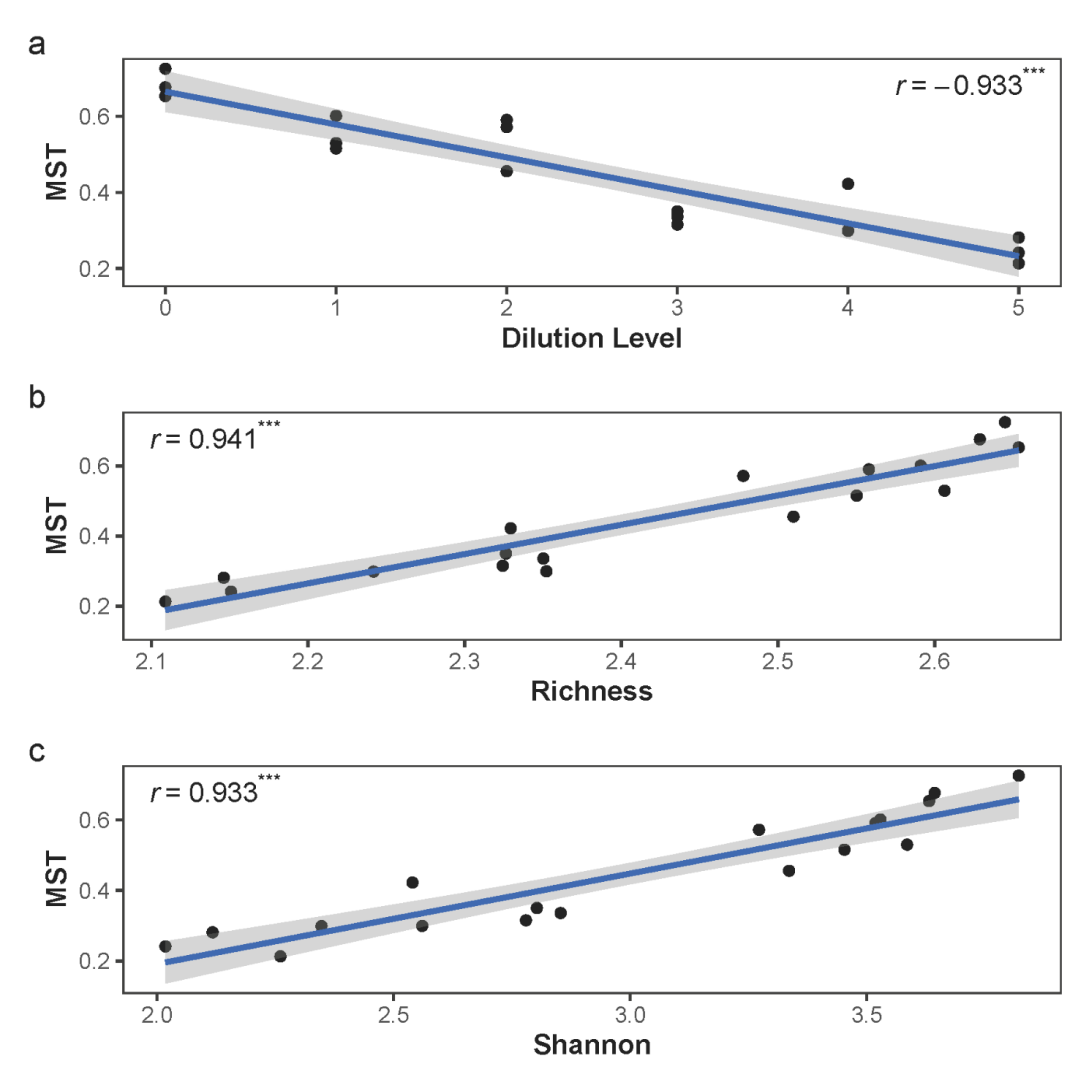
The relationship between modified stochasticity ratio (MST) and (a) dilution level, (b) species richness, and (c) the Shannon index in the microcosm study. MST represents the relative importance of stochastic process in community assembly. MST smaller than 0.5 represents stronger deterministic processes than stochastic processes and MST larger than 0.5 represents stronger stochastic processes than deterministic processes. Dilution level equals to the logarithmically transformed dilution factor based on 10. Species richness is logarithmically transformed based on 10 before the calculation of Pearson’s correlation. The lines show the result of linear regression and the shaded areas represent 95% confidence intervals. Here, *r* represents the Pearson’s correlation and *** represents *P* < 0.001.

MST decreased with increasing dilution level, suggesting stronger deterministic processes at a higher dilution level (Pearson’s *r* = −0.933, *P* < 0.001; Fig. 1a). We observed a negative correlation between MST and species richness (Pearson’s *r* = 0.941, *P* < 0.001; Fig. 1b) and the Shannon index (Pearson’s *r* = 0.933, *P* < 0.001; Fig. 1c). We also observed a higher MST in rare species than in abundant species (Figure S4), indicating that rare species community assembly were more driven by stochastic processes than abundant species.

**Fig. 2:**
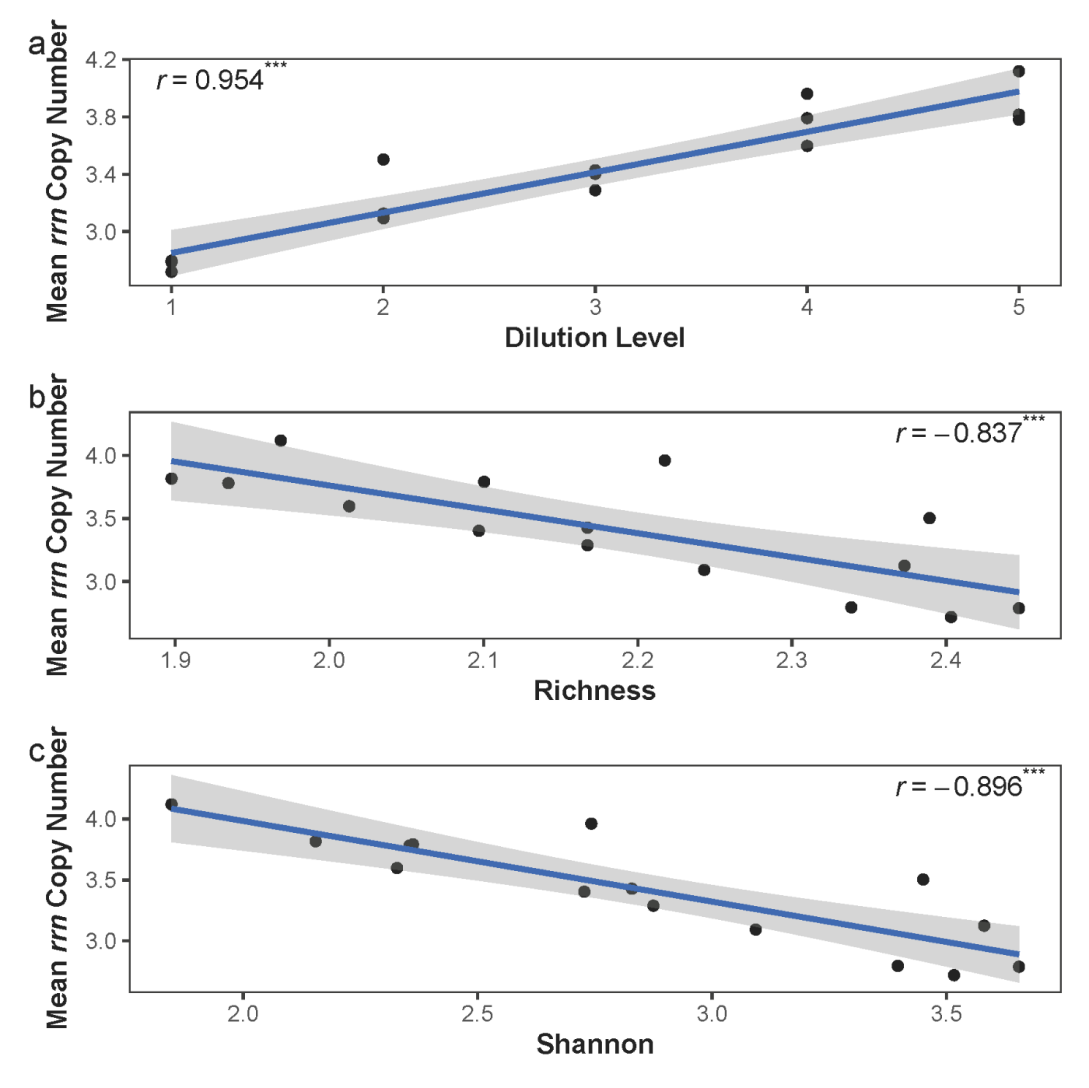
The relationship between mean *rrn* copy number and (a) dilution level, (b) species richness, and (c) Shannon index in the microcosm study. Mean *rrn* copy number is the abundance-weighted mean rRNA operon copy number. Dilution level equals to the logarithmically transformed dilution factor based on 10. Species richness is logarithmically transformed based on 10. The lines show the result of linear regression and the shaded areas represent 95% confidence intervals. Here, *r* represents the Pearson’s correlation and *** represents *P* < 0.001

We predicted the *rrn* copy number for each OTU to classify different OTUs into copiotrophs and oligotrophs. The species with higher *rrn* copy numbers tended to be more frequent in diluted communities (generalized linear model: slope = 0.041, *P* < 0.001), so fast growth rates might support persistence in diluted communities after regrowth. At the community level, the mean *rrn* copy number increased with a higher dilution level (Pearson’s *r* = 0.954, *P* < 0.001; Fig. 2a) and decreased with higher species richness (Pearson’s = − 0.837, *P* < 0.001; Fig. 2b) and the Shannon index (Pearson’s *r* = − 0.896, *P* < 0.001; Fig. 2c). Thus, MST decreased with higher mean *rrn* copy number (Pearson’s *r* = − 0.792, *P* < 0.001; Figure S5). These results suggested that the copiotrophs were selected under higher dilution levels.

**Fig. 3:**
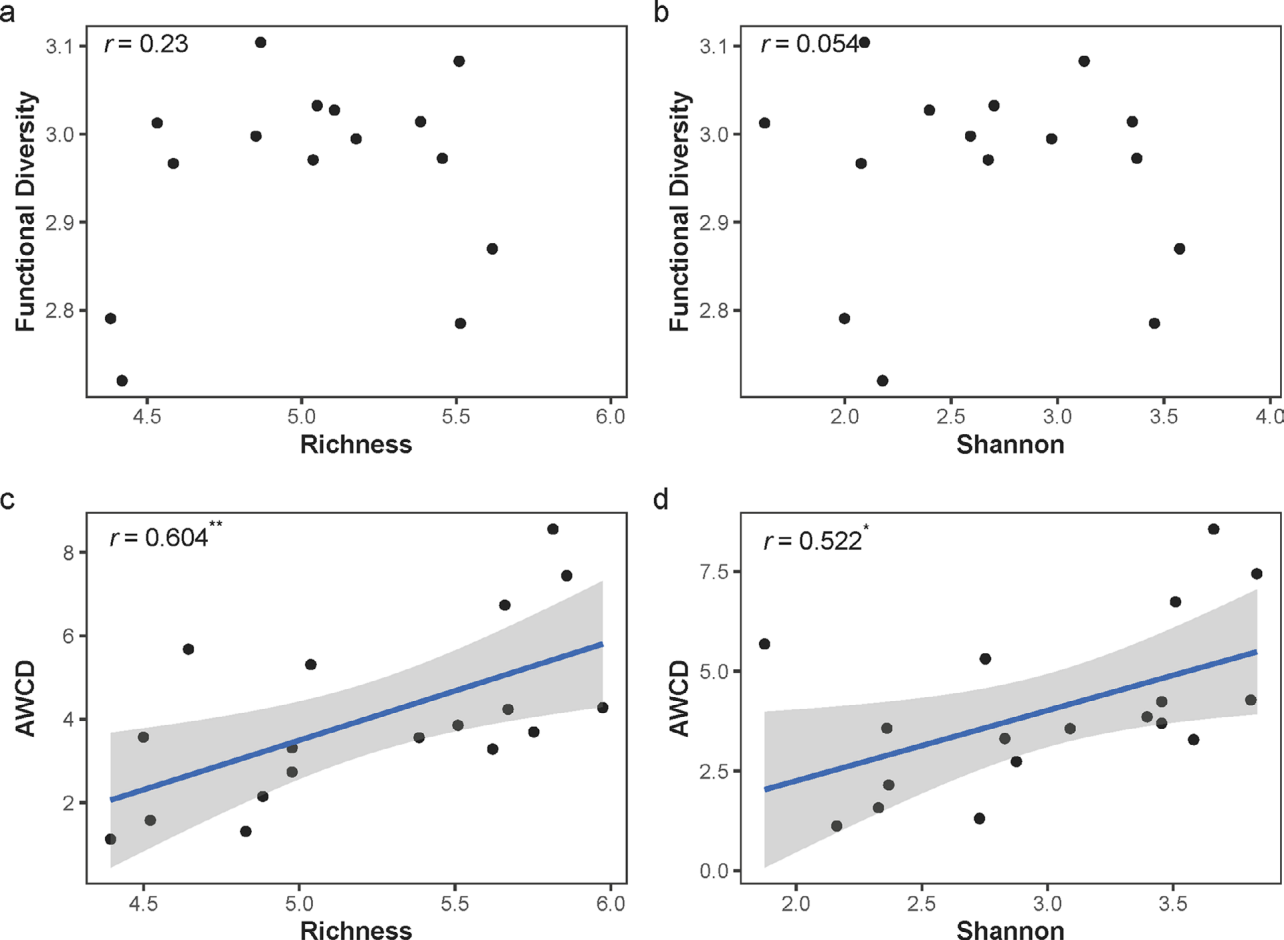
The relationships between taxonomic diversity and ecosystem functions in the microcosm study. The relationship between functional diversity and (a) species richness and (b) Shannon index. The functional diversity represents the diversity of carbon substrate utilization using Biolog Ecoplate™ when the average well color development is closest to 0.5. The relationship between average well color development (AWCD) and (c) species richness as well as (d) Shannon index. The AWCD is measured three days’ culture and further corrected by initial cell density. Richness is logarithmically transformed based on 10. The lines show the result of linear regression and the shaded areas represent 95% confidence intervals. *r* represents the Pearson’s correlation and *** represents *P* < 0.001, ** represents *P* < 0. 01, and * represents *P* < 0.05.

We used Biolog EcoPlate™ to measure the carbon utilization ability as an ecosystem function. The functional diversity (the Shannon index of utilized carbon) and AWCD were used here. Functional diversity was not affected by the loss of species richness (Pearson’s *r* = 0.151, *P* = 0.260; Fig. 3a) or the Shannon index (Pearson’s *r* = 0.054, *P* = 0.831; Fig. 3b). When considering the AWCD corrected by cell density, we found a positive relationship between ecosystem functions and species richness (Pearson’s *r* = 0.610, *P* = 0.007; Fig. 3c) as well as the Shannon index (Pearson’s *r* = 0.522, *P* = 0.026; Fig. 3d).

### Meta-analysis

We collected 82 DTE experiments from 26 different articles with prokaryotic composition information using the high-throughput amplicon sequencing method (Table S1). In most DTE experiments, species richness (z_++_ = − 1.29, *P* < 0.001; Figure S6a) and the Shannon index (z_++_ = − 1.24, *P* < 0.001; Figure S6b) decreased with higher dilution level. The DTE experiments that species richness didn’t significantly decrease with dilution levels were removed in later analysis because they were likely to be contaminated or the diversity of the initial microbial community was too small to get a good dilution result. Finally, 70 DTE experiments remained for later analyses.

We examined the changes in the relative abundance of each phylum induced by dilution. The dominant phyla/classes were different in each habitat. We focused on soil ecosystems that had the largest experimental numbers in the meta-analysis. We found that the relative abundance of *Betaproteobacteria* significantly increased with dilution levels among different studies (z_++_ = 0.357, *P* < 0.001; Table S2).

**Fig. 4:**
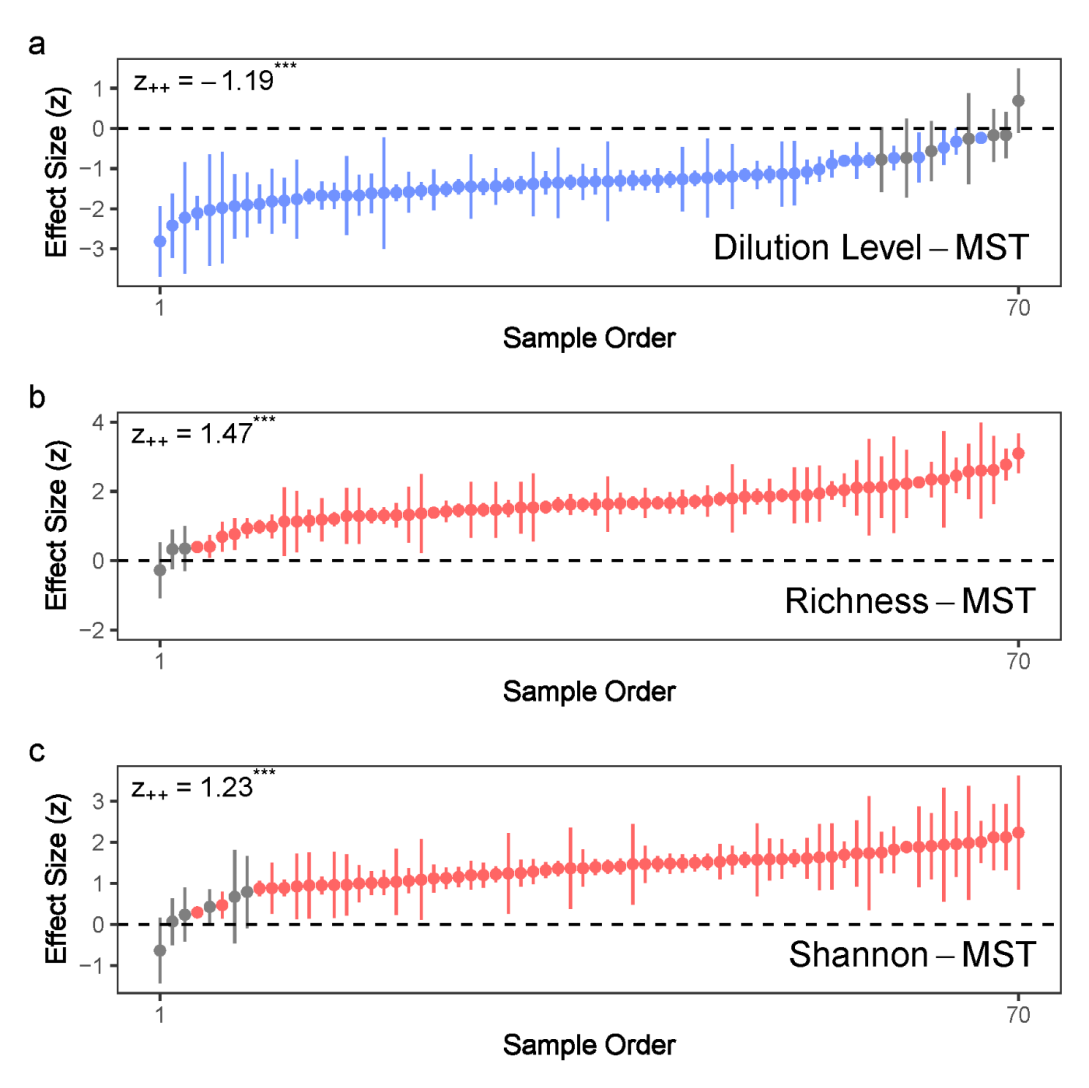
The correlation between modified stochasticity ratio (MST) and (a) dilution level, (b) species richness or (c) Shannon index for each experiment in the meta-analysis. The Pearson’s correlation is first calculated and transformed into effect size using Fisher’s z transformation. Dilution level equals to the logarithmically transformed dilution factor based on 10. Species richness is logarithmically transformed based on 10 before the calculation of Pearson’s correlation. The points (with 95% confidence intervals) represent effect sizes in different experiments and are given in increasing order. Red color represents the effect sizes significantly larger than 0, blue color represents the effect sizes significantly smaller than 0 and grey color represents effect sizes having no significant difference with 0. z_++_ is the estimate of mean effect size using meta regression and *** represents *P* < 0.001 using meta-analysis model

MST decreased toward higher dilution levels (z_++_ = − 1.28, *P* < 0.001; Fig. 4a), which resulted in a positive relationship between MST and species richness (z_++_ = 1.47, *P* < 0.001; Fig. 4b) as well as the Shannon index (z_++_ = − 1.23, *P* < 0.001; Fig. 4c). It indicates stronger deterministic processes toward higher dilution levels. The communities of rare species had significantly higher MST than the communities of abundant species (Figure S7). Community assembly of rare species are more driven by stochastic processes than that of abundant species.

**Fig. 5:**
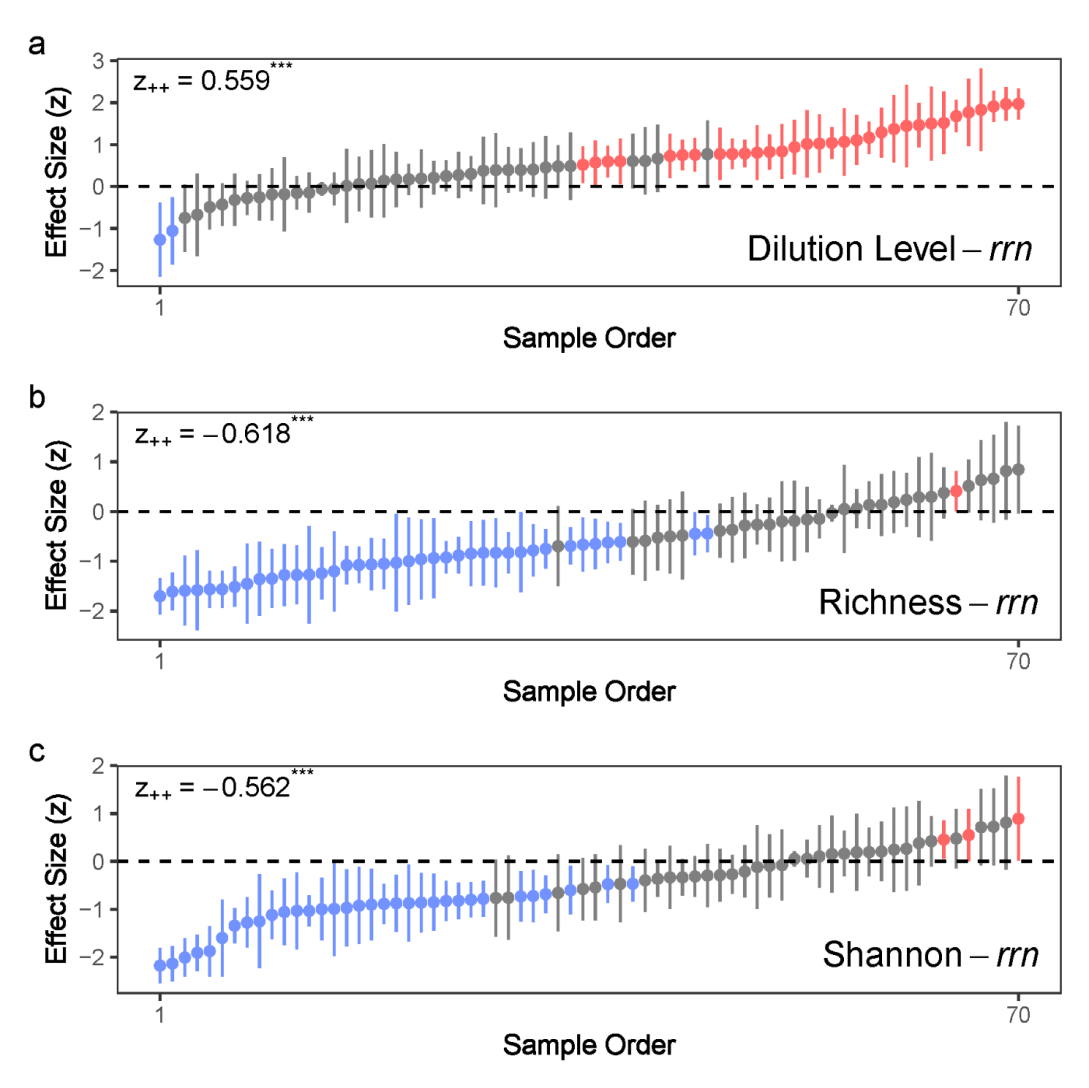
The correlation between mean *rrn* copy number and (a) dilution level, (b) species richness or (c) Shannon index for each experiment in the meta-analysis. The Pearson’s correlation is first calculated and transformed into effect size using Fisher’s z transformation. Dilution level equals to the logarithmically transformed dilution factor based on 10. Species richness is logarithmically transformed based on 10 before the calculation of Pearson’s correlation. The points (with 95% confidence intervals) represent effect sizes in different experiments and are given in increasing order. Red color represents the effect sizes significantly larger than 0, blue color represents the effect sizes significantly smaller than 0 and grey color represents effect sizes having no significant difference with 0. z_++_ is the estimate of mean effect size using meta regression and *** represents *P* < 0.001 using meta-analysis model

The OTUs with higher *rrn* copy numbers had significantly higher frequency of occurrence in diluted communities in 63.8% of the DTE experiments (Table S3). At the community level, the dilution level increased the mean *rrn* copy number (z_++_ = 0.559, *P* < 0.001; Fig. 5a), and the mean *rrn* copy number was negatively correlated with species richness (z_++_ = − 0.618, *P* < 0.001; Fig. 5b) and the Shannon index (z_++_ = − 0.562, *P* < 0.001; Fig. 5c). The MST within the dilution level was also negatively correlated with the mean *rrn* copy number (z_++_ = − 0.552, *P* < 0.001; Figure S8).

**Fig. 6:**
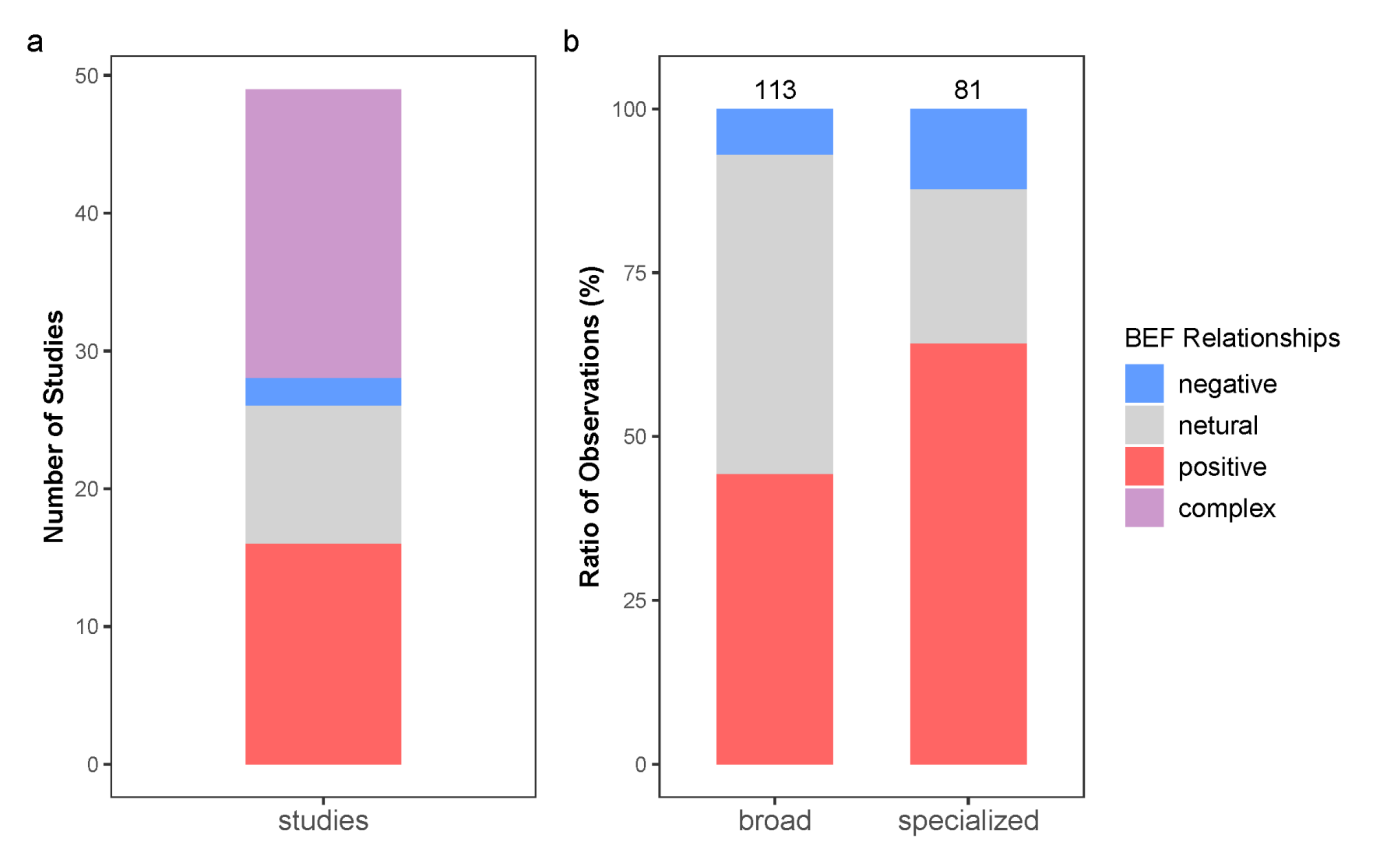
The biodiversity-ecosystem functions relationships in (a) different studies and (b) different observations. (a) The number of studies reporting positive, negative, neutral and complex BEF relationships respectively. Here, the complex BEF relationships represent the studies which report more than one kind of BEFs (positive, neutral and negative) using different functions or the same function under different conditions. (b) The ratio of positive, neutral and negative BEF relationships considering broad functions and specialized functions respectively. The number above each column showed the number of observations for broad functions and specialized functions. Different observations of ecosystem functions are separated into broad functions and specialized functions. Broad functions are functions thought to be carried out by most microbes, including bacterial activities and degradations of labile carbon. Specialized functions are functions thought to be carried out only by specific microbes, including degradation of inert carbon, nitrogen cycling and other elementary cycling

To further explore the BEF relationships, we also collected 66 studies using DTE to study BEF relationships. Going through different BEF relationships found in different studies, we found that 37.9%, 10.6% and 3.0% of studies reported positive, neutral and negative BEF relationships respectively (Fig. 6a). Approximately 48.5% of studies reported complex BEF relationships, which contain more than one kind of BEF relationships (positive, negative or neutral) when different ecosystem functions or the same ecosystem functions under different conditions were studied (Table S6). When we classified functions into broad and specialized functions, the BEF relationships showed a contrasting pattern (Fig. 6b). Broad functions showed higher ratio of neutral and lower ratio of positive BEF relationships than specialized functions.

## Discussion

DTE, as an important method to manipulate microbial diversity, is widely used to explore BEF relationships in different ecosystems and significantly promotes our understanding of the importance of microbial diversity [10, 17, 24]. However, most studies focused on taxonomic diversity changes caused by DTE. Here, we observed the selection of copiotrophs and the reduction of rare species as well as enhanced deterministic processes in microbial community assembly towards higher dilutions using microcosm study and meta-analysis. These change in community structure may result in more complex BEF relationships in microbial communities when broad microbial functions are considered. We also found more neutral and less positive BEF relationships in broad functions than specialized functions in DTE studies.

### Selection of copiotrophs is responsible for stronger deterministic processes at a higher dilution level

Deterministic processes have been demonstrated to become stronger at a higher dilution level because of reduced microbial diversity [19, 24]. However, the communities with low diversity are not necessarily dominated by deterministic processes, as some studies found stronger stochastic processes than deterministic processes in communities with low diversity rather communities with high diversity [48, 54]. We highlight the contributions of loss of rare species and selection of copiotrophs to community assembly. Community assembly of rare species is more commonly driven by stochastic processes than that of abundant species [55], which are also observed in this study (Figure S4; Figure S6). Loss of rare species weakens the stochastic processes, while selection of copiotrophs strengthens deterministic processes. At a higher dilution level, bacteria may spend a longer time in regrowth, i.e., obtaining biomass/abundance similar to undiluted communities [10]. During regrowth, copiotrophs could outcompete oligotrophs like what happening in the early stage of primary succession [33]. Similarly, Abreu et al. found that the copiotrophs are more likely to outcompete the oligotroph at higher dilution rates using 2- to 5-species coculture experiments [56]. This could be owing to the fast growth rates which could favor copiotrophs to quickly occupy the empty niche caused by disturbance [33, 57] or dilution [56]. Therefore, OTUs with high *rrn* copy numbers are more likely to persist in highly diluted microbial communities. *Betaproteobacteria*, which are thought to be copiotrophs in both freshwater [58] and soil ecosystems [28, 59] were found to increase with dilution level in both microcosm and the meta-analysis studies. The strong deterministic processes in communities with low diversity at high dilution level could be negative to ecosystems if the limited species selected by deterministic processes are not the ones carrying out important ecosystem functioning [23].

### Loss of rare species causes the loss of specialized functions in DTE

In real scenarios, not all species face the same danger of extinction [60]. Species with low abundance in natural ecosystems are more likely to be lost due to different stressors, habitat fragmentation and drift [61]. DTE is thought to remove rare species, meeting the need for rare species loss [61, 62] and make DTE a popular method [10, 17]. Rare species making up the majority in natural communities play an essential role in ecosystem functioning [63]. Many specialized functional genes are carried by rare species in microbial communities such as the sulphate reduction or phenanthrene degradation [47, 64, 65]. Thus, the low “redundancy” of specialized functional genes makes these functional performances more vulnerable to diversity loss [66]. For example, the abilities of chitin and cellulose degradation [67], xenobiotic carbon degradation [68, 69], N_2_O reduction [70], sulfate reduction [71], and Fe(III) reduction [72] are easily lost within a few steps of 10-fold dilution. It explains our observation that microbial specialized functions were impaired by biodiversity loss in most DTE studies (Fig. 4). Furthermore, the microbial specialized functions are better used for defining BEF relationships in DTE experiments as well as in field BEF observations.

### Different ratios of copiotrophs might change BEF relationships

Biodiversity not only includes the number of existing taxa, but also includes functional and phylogenetic information [10, 73]. Functional diversity is well believed to be a better predictor for ecosystem functioning than simple taxonomic richness [73, 74]. For microorganisms, some functional traits at the genome level, such as the *rrn* copy number studied in this study, are effective in predicting species performance [75]. When those traits are applied at the community level, they are potential to give better predictions for the differences in microbial functional performance [76].

The biodiversity effect can be divided into the complementarity effect and the sampling effect (also called as selection effect) [77]. The complementarity effect means that different species could enhance ecosystem functions through niche separation or positive interactions [77]. The sampling effect means that the dominant species may have a strong effect on ecosystem functions and that the productive species are more likely to be present in diverse communities [77]. If the dominant species strongly favor a certain ecosystem function, the functional performance could be high even in a low-diversity community [78]. In the DTE experiments, the higher ratio of copiotrophs in low diversity communities could result in higher functional performance through strong sampling effects. Compared to oligotrophs, copiotrophs adapt better to resource-rich conditions with faster growth rates and quick response to substrate addition [28]. For example, copiotrophs could utilize carbon substrates more widely and quickly than oligotrophs [28, 79]. This could explain why we found neutral BEF relationships in the diversity of carbon substrates (Fig. 3a, b). At the microbial community’s level, highly diluted communities may have a higher relative abundance of broad function-related genes than less diluted communities in DTE experiments [19]. Thus, when considering broad functions, the sampling effect could outweigh the complementarity effect and lead to a neutral and even negative BEF relationship.

### Implications for DTE

The *rrn* copy number is well known to vary from 1 to 15 in bacterial genomes and 1 to 4 in archaeal genomes [80]. The species with a high *rrn* copy number could result in a high abundance in sequencing, although with a low cell number. To resolve this case, a pipeline to remove this bias has been built up [45]. We observed a significant change in the *rrn* copy number after dilution, which means that the *rrn* copy number should be used to correct the abundance data and obtain the true bacterial cell number in later DTE studies.

Similarly, a correction is also needed for the result of quantitative polymerase chain reaction (qPCR). In dilution-to-extinction studies, the most widely used method to monitor the regrowth of microbial biomass is qPCR [9, 24, 81]. Most of the studies used it to represent the bacterial biomass directly except the study by Domeignoz-Horta et al. [81]. This method may not make a significant change in the natural community, where the ratio of copiotrophs is quite low [82, 83] and their mean *rrn* copy numbers are close to others. As we proved, the mean *rrn* copy number increased with dilution level and, thus, the direct use of qPCR may be inaccurate to represent biomass recovery. It is important to use mean *rrn* copy number to correct the result of qPCR or using other methods, such as cell density using a flow cytometer [10] and microbial carbon [84] to quantify the microbial biomass.

## Conclusion

The dilution-to-extinction experiments involve complex microbial ecological processes. We found that deterministic processes become important with increasing dilution levels because of the selection of copiotrophs and the loss of rare species. The structural shift from oligotroph dominance to copiotroph dominance caused by dilution-to-extinction can change functional performance and lead to more complex BEF relationships in DTE studies, especially for broad functions. Microbial specialized functions could be better used for quantifying BEF relationships in DTE experiments as well as in field BEF observations. In addition, the selection of copiotrophs may cause a higher mean *rrn* copy number and make qPCR, if not corrected, ineffective in representing the true biomass. Our findings are helpful for future studies exploring microbial BEF relationships.

## Electronic supplementary material

Below is the link to the electronic supplementary material.

Supplementary Material 1

## Data Availability

The sequencing data of the microcosm study are available in the National Center for Biotechnology Information with the Sequence Read Archive bioproject number PRJNA864105. The OTU table and related processing code of the meta-analysis could be found in figshare (10.6084/m9.figshare.21586701).

## Abbreviations

DTE: dilution-to-extinction
BEF: biodiversity-ecosystem function
*rrn*: ribosomal RNA operon
OTU: operational taxonomic unit
MST: modified stochasticity
AWCD: average well color development
qPCR: quantitative polymerase chain reaction
