# Supplementary material for: The selection of copiotrophs may complicate biodiversity-ecosystem functioning relationships in microbial dilution-to-extinction experiments
Source: Environ Microbiome. 2023 Mar 17;18:19. doi: 10.1186/s40793-023-00478-w (PMC10024408; doi:10.1186/s40793-023-00478-w)
Supplement: Supplementary file 1 — Supplementary Material 1 [file 40793_2023_478_MOESM1_ESM.docx]

**On Line Supplementary Information For**

**The selection of copiotrophs may complex biodiversity-ecosystem functioning relationships in microbial dilution-to-extinction experiments**

Zhendu Mao ^1, 3^, Zifan Zhao ^1, 3^, Jun Da^1, 4^, Ye Tao^1, 3^, Huabing Li ^1^, Biying Zhao ^5^, Peng Xing ^1^, Qinglong Wu ^1, 2,^ *****

^1^ State Key Laboratory of Lake Science and Environment, Nanjing Institute of Geography and Limnology, Chinese Academy of Sciences, Nanjing 210008, China

^2^ Center for Evolution and Conservation Biology, Southern Marine Sciences and Engineering Guangdong Laboratory (Guangzhou), Guangzhou 511458, China

^3^ University of Chinese Academy of Sciences, Beijing 100049, China

^4^ College of Life Science, Anhui Normal University, Wuhu 241002, China

^5^ International Genome Center, Jiangsu University, Zhenjiang 212013, China

*Authors for correspondence. Qinglong Wu, E-mail: [qlwu@niglas.ac.cn](mailto:qlwu@niglas.ac.cn). Phone: +86-25-86882107

**Key Words:** Dilution-to-extinction; community assembly processes; biodiversity-ecosystem functioning;


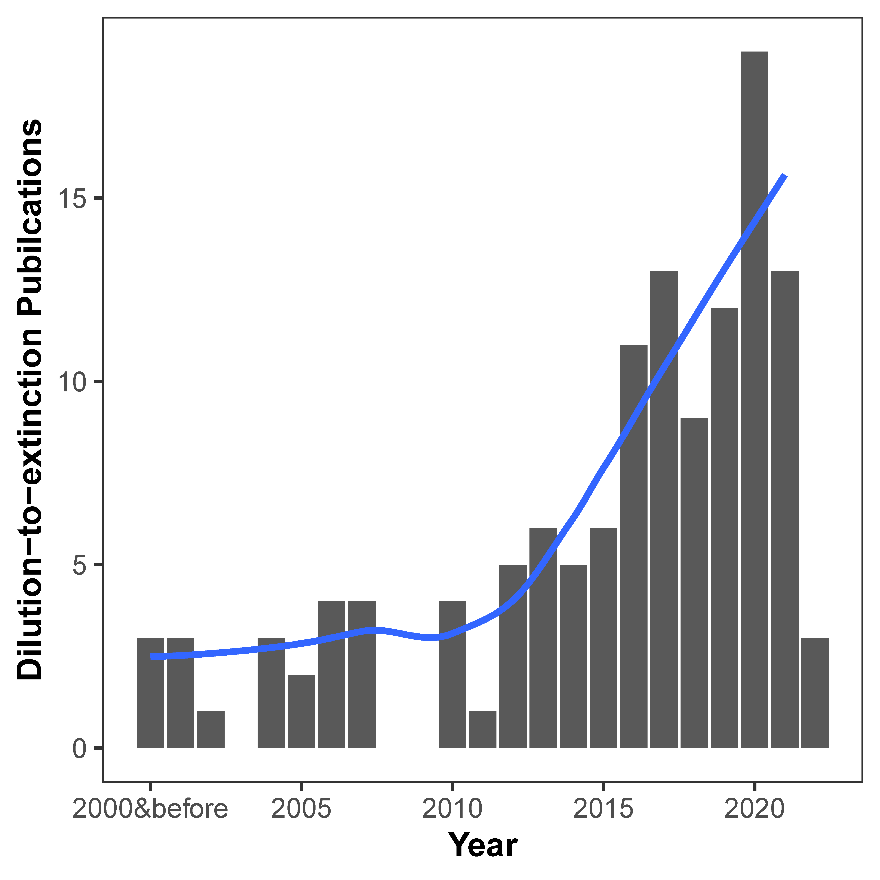


**Figure S1** The number of collected dilution-to-extinction publications in different years. These publications describe the function and/or composition shift using dilution-to-extinction. The blue line shows the trend in the number of publications.


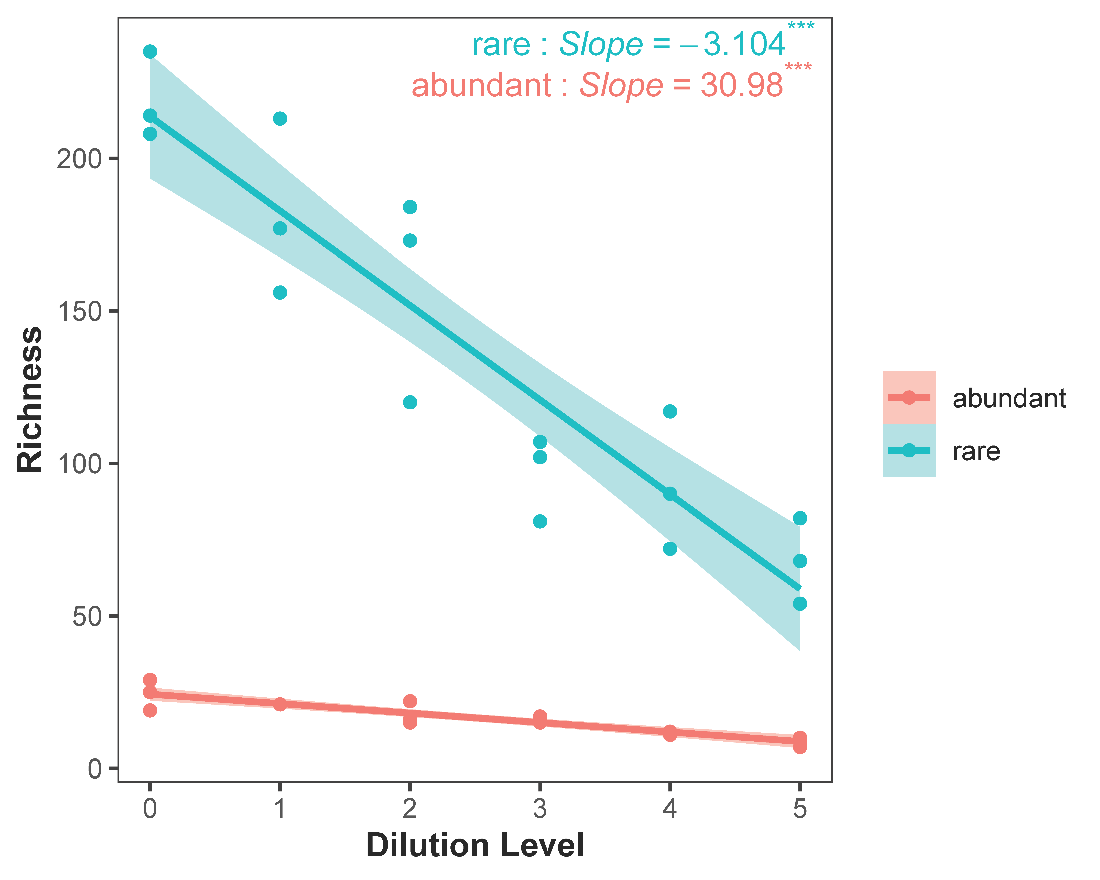


Figure S2 The species richness of abundant species and rare species in each dilution level in the microcosm study. Dilution level equals to the log-transformed dilution factor. The lines show the result of linear regression and the shaded areas represent 95% confidence intervals. The abundant species mean the species with relative abundance higher than 1% and rare species mean the species with relative abundance lower 0.1%.


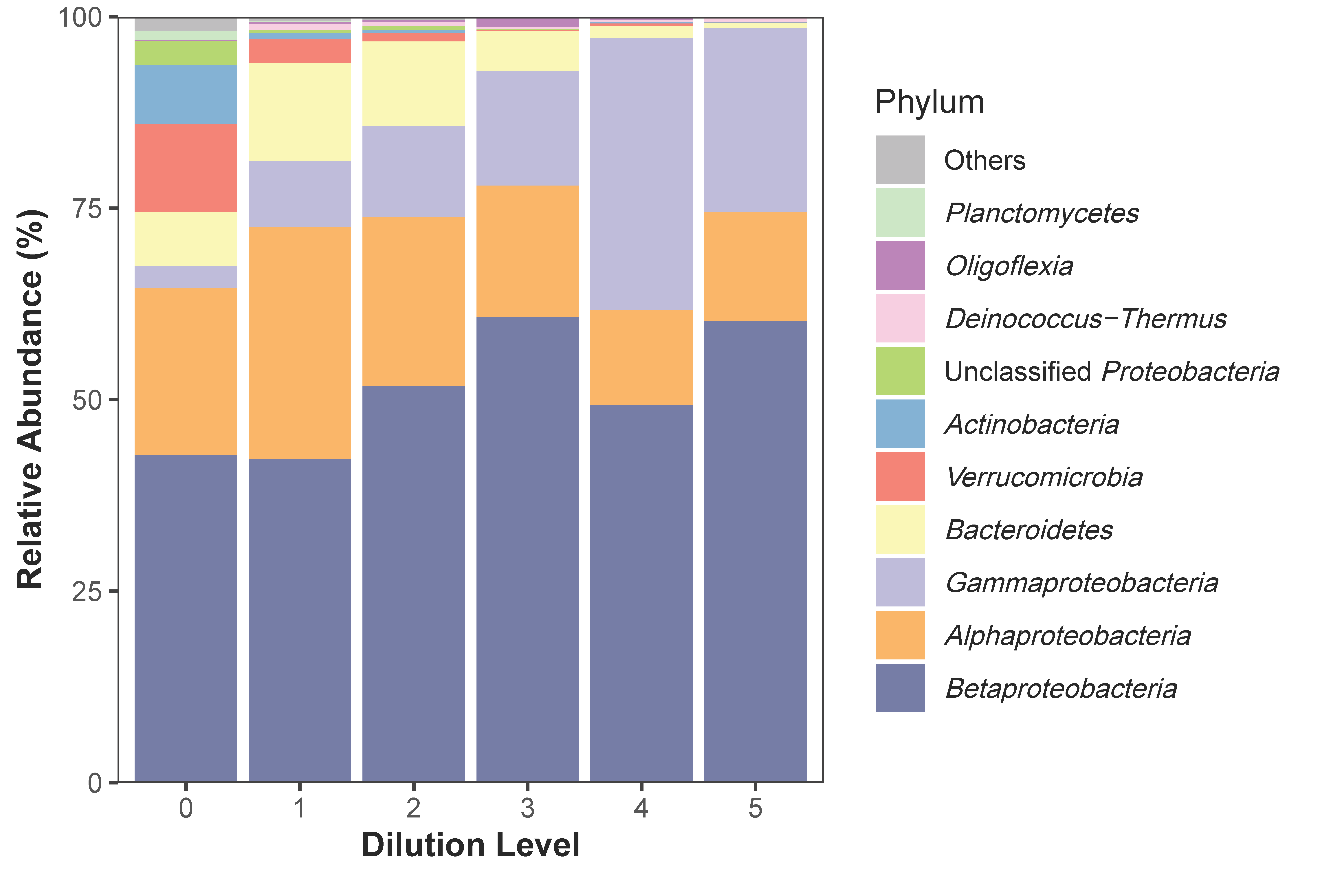


Figure S3 The relative abundance of abundant phyla/classes in different dilution level in the microcosm study. Here, the phylum *Proteobacteria* are further separated into *Alphaproteobacteria*, *Betaproteobacteria*, *Gammaproteobacteria*, and Unclassified *Proteobacteria*.


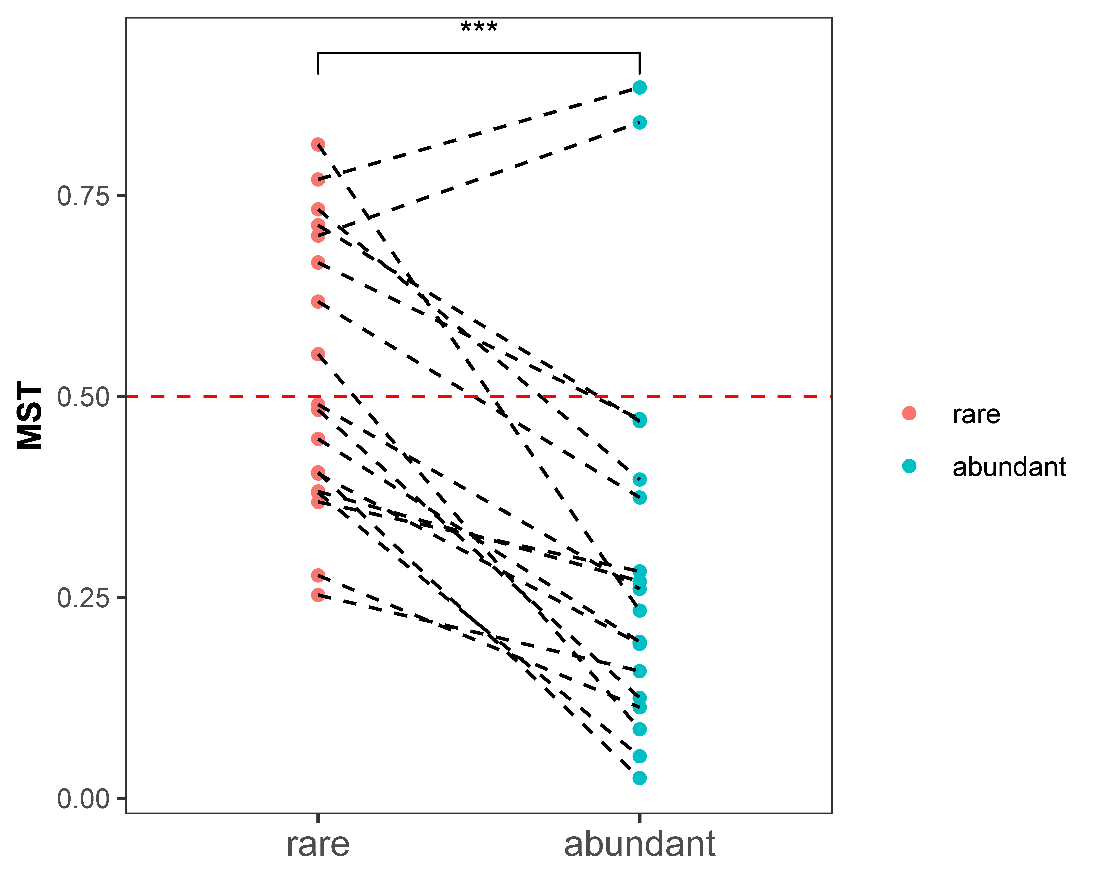


**Figure S4** The modified stochasticity (MST) among abundant species and rare species in the microcosm study. The abundant species mean the species with relative abundance higher than 1% and rare species mean the species with relative abundance lower 0.1%. The MST difference between rare species and abundant species was tested using paired t-test and *** represents *P* < 0.001.


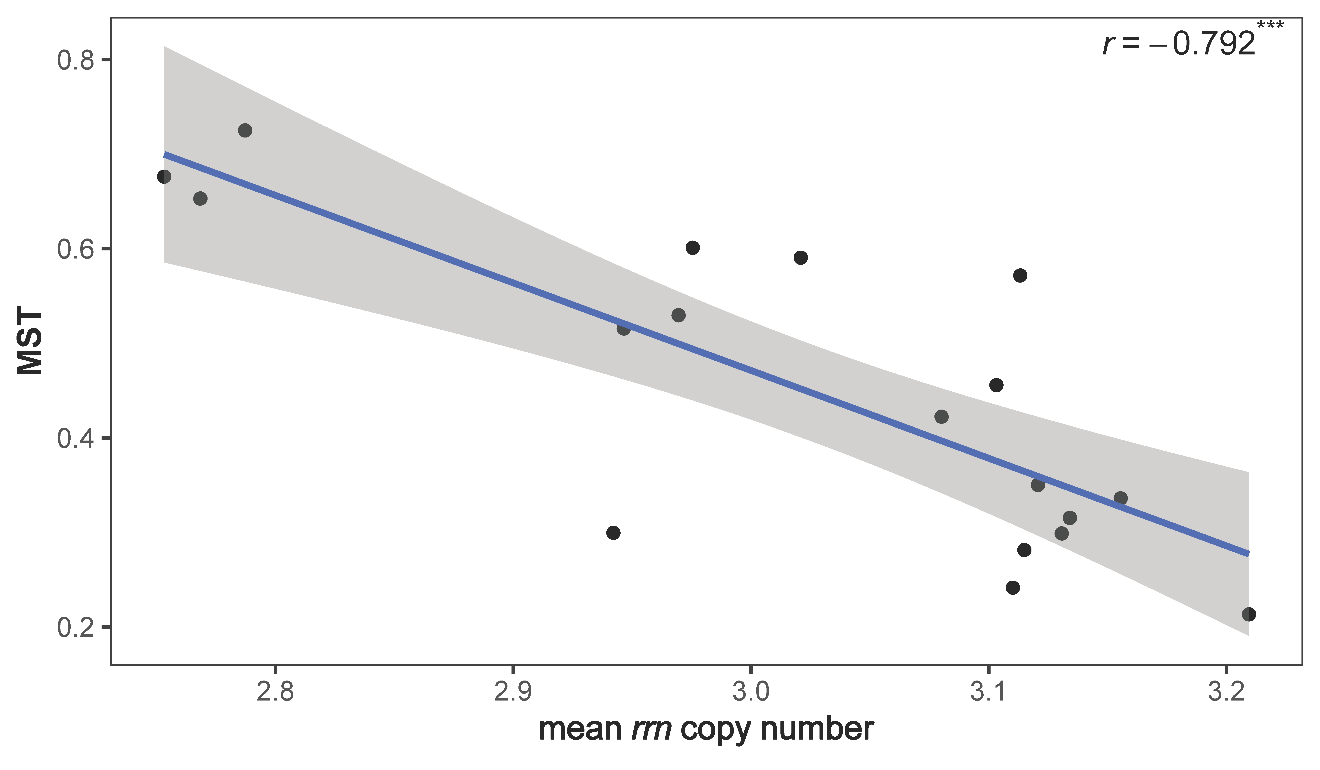


Figure S5 The relationship between modified stochasticity ratio (MST) and mean *rrn* copy number in the microcosm study. The lines show the result of linear regression and the shaded areas represent 95% confidence intervals. *r* represents the Pearson’s correlation and *** represents *P* < 0.001.


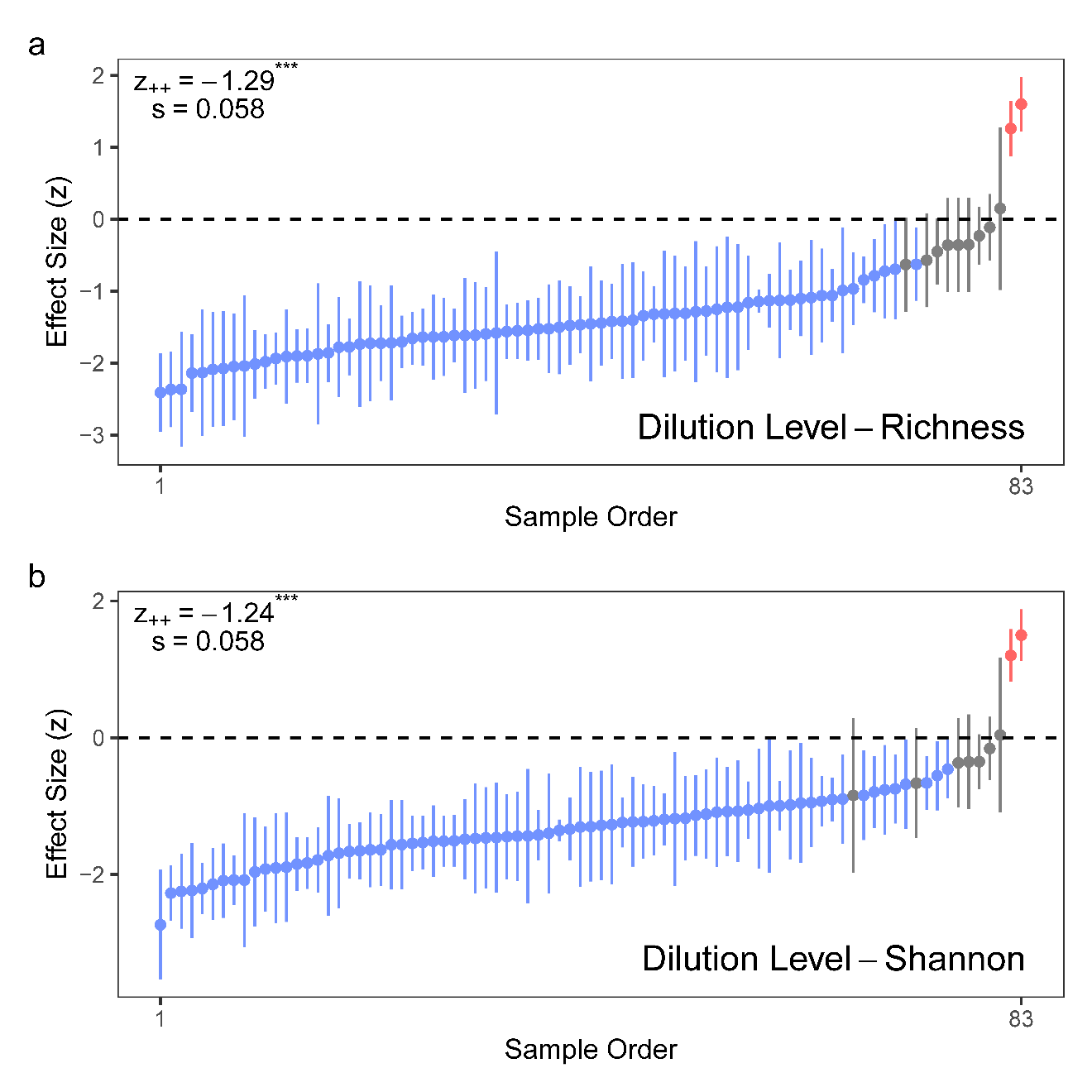


**Figure S6** The correlation between dilution level and (a) richness and (b) Shannon index change in the meta-analysis. The Pearson’s correlation is first calculated and transformed into effect size using Fisher’s z transformation. Dilution level equals to the logarithmically transformed dilution factor based on 10. Species richness is logarithmically transformed based on 10 before the calculation of Pearson’s correlation. Effect sizes (95% confidence intervals) are given in increasing order. Red color represents the effect sizes significantly larger than 0, blue color represents the effect sizes significantly smaller than 0 and grey color represents effect sizes having no significant difference with 0. z_++_ is the estimate of mean effect size using meta regression and *** represents *P* < 0.001 using meta-analysis model.

The experiments with non-significant correlation between dilution level and species richness are removed in later analysis.


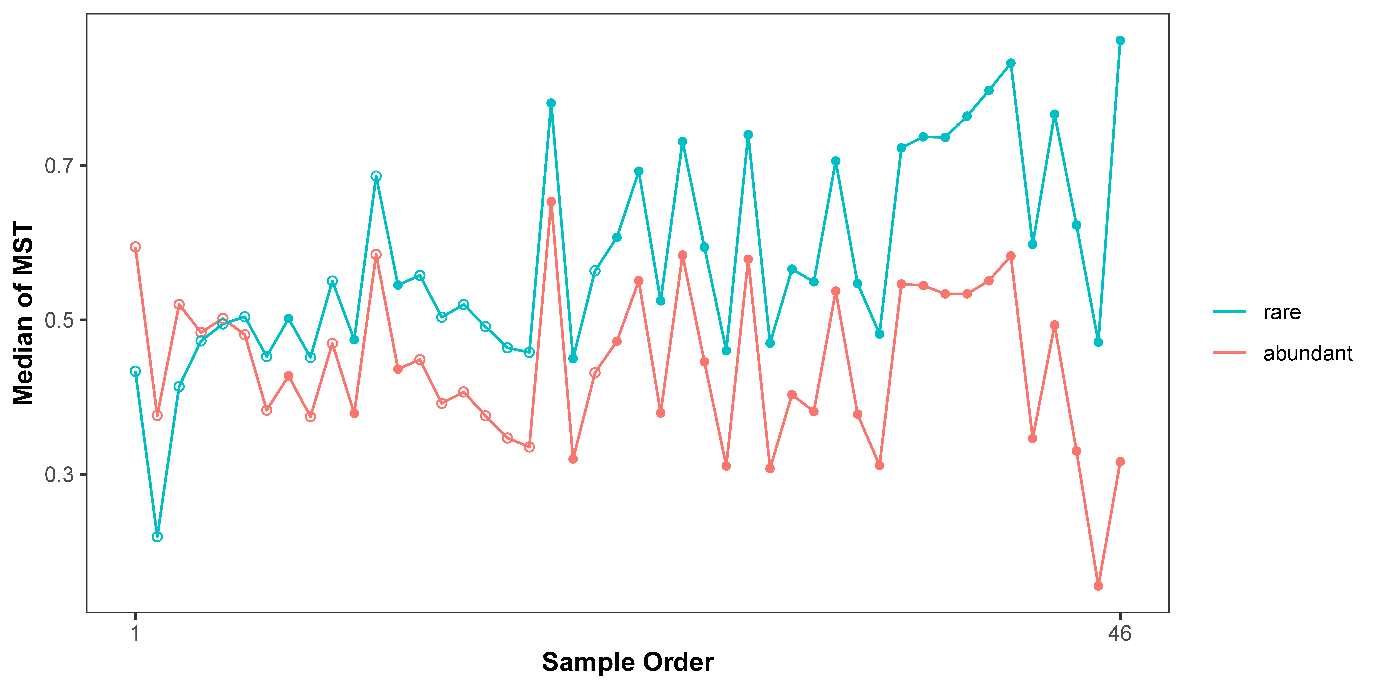


**Figure S7** The mean of modified stochasticity ratio (MST) in each experiment for rare species and abundant species communities in the meta-analysis. The samples are given in increasing order of the MST differences between rare species (relative abundance < 0.1%) and abundant species (relative abundance > 1%). 24 experiments are discarded because they fail to calculate the MST for rare species and/or abundant and don’t have sufficient data for statistical analysis. The MST difference between rare species and abundant species in each experiment was tested using paired t-test. Open circles represent that there is no significant difference between rare species and abundant species and closed circles represent that there is significant difference between rare species and abundant species.


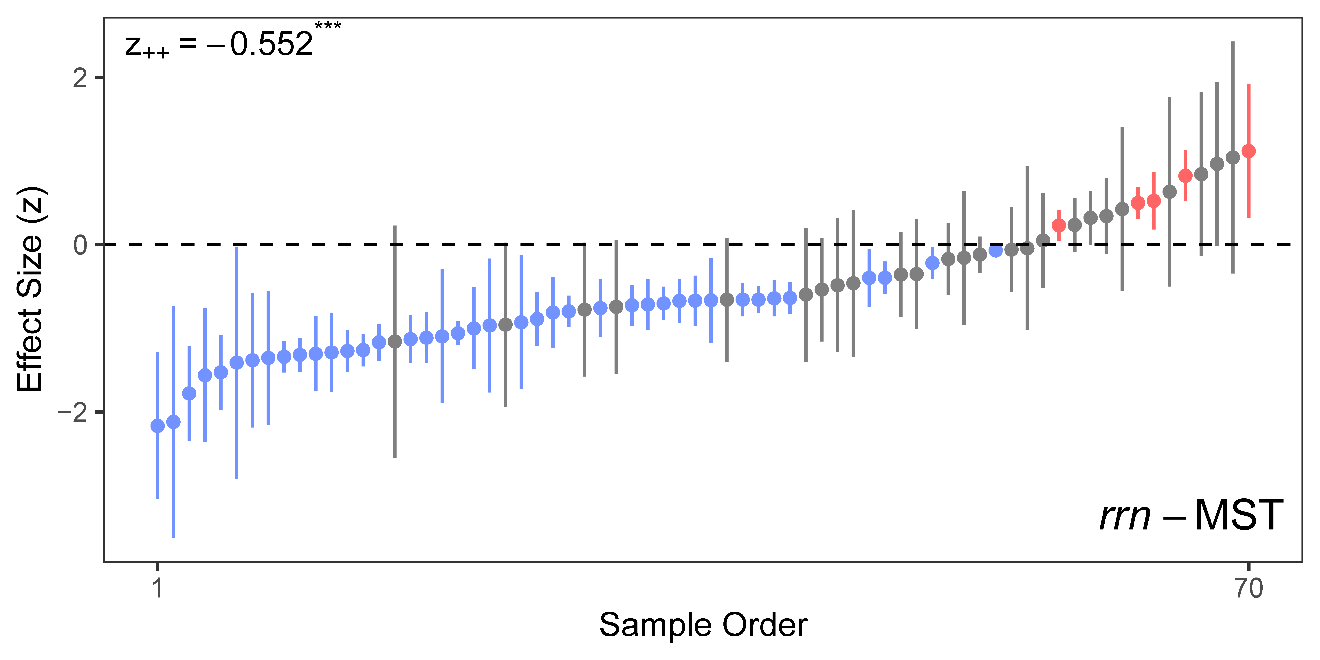


Figure S8 The relationship between modified stochasticity ratio (MST) and mean *rrn* copy number in the meta-analysis. Red color represents the effect sizes significantly larger than 0, blue color represents the effect sizes significantly smaller than 0 and grey color represents effect sizes having no significant difference with 0. z_++_ is the estimate of mean effect size using meta regression and *** represents *P* < 0.001 using meta-analysis model.

**Table S1** The list of publications whose community information was reanalyzed in this study

| Publication | DOI | Number of experiments | Accession in public database or website |
| --- | --- | --- | --- |
| Albright_PO | 10.1371/journal.pone.0224641 | 2 | PRJNA601499 |
| Arruda_Mycorrhiza | 10.1007/s00572-021-01044-3 | 1 | PRJEB42785 |
| Calderon_ISME | 10.1038/ismej.2016.86 | 4 | PRJNA281221 |
| Chen_EI | 10.1016/j.envint.2020.105766 | 1 | PRJNA563500 |
| Chen_ESPR | 10.1007/s11356-019-05781-z | 1 | SRP149305 |
| Costa_SBB | 10.1016/j.soilbio.2020.107893 | 8 | PRJNA563456 |
| Daval_MB | 10.1111/1751-7915.13634 | 1 | PRJEB36457 |
| Domeignoz_NC | 10.1038/s41467-020-17502-z | 4 | PRJNA556439 |
| Ernakovich_Biogeochemistry | 10.1007/s10533-020-00736-w | 2 | PRJNA689861 |
| Feng_ME | 10.1111/mec.14356 | 3 | SRP091356 |
| Ferreira_ME | 10.1007/s00248-020-01502-z | 4 | PRJNA599204 |
| Hol_Ecology | 10.1890/14-2359.1 | 5 | PRJEB5295 |
| Howard_FEMSML | 10.1093/femsle/fnx092 | 1 | SRP101475 |
| Maron_AEM | 10.1128/AEM.02738-17 | 1 | PRJEB19513 |
| Saghai_ASE | 10.1016/j.apsoil.2021.104224 | 6 | PRJNA637634 |
| Saavedra_CJM | 10.1139/cjm-2019-0412 | 5 | PRJNA543329 |
| Stadler_MB | 10.1111/1751-7915.12870 | 2 | PRJNA319442 |
| Tardy_EMR | 10.1111/1758-2229.12126 | 1 | PRJEB4260 |
| Yan_ISME | 10.1038/ismej.2016.108 | 5 | PRJEB12988 |
| Yu_ISME | 10.1038/s41396-019-0356-5 | 1 | PRJNA477654 |
| Zegeye_AEM | 10.1128/mSystems.00055-19 | 18 | <https://doi.org/10.25584/data.2019-02.700/1506698> |
| Zehavi_FiM | 10.3389/fmicb.2018.01999 | 3 | SRP153190 |
| Zha_PO | 10.1371/journal.pone.0155239 | 1 | PRJNA272853 |
| Zhang_PO | 10.1371/journal.pone.0126962 | 1 | PRJNA278816 |
| Zhang_EST | 10.1021/acs.est.8b06044 | 2 | PRJNA427844 |
| Zhang_SBB | 10.1016/j.soilbio.2016.04.017 | 1 | PRJNA280782 |

**Table S2** The generalized linear models between *rrn* copy number and frequency of occurrences in diluted communities with a Poisson distribution.

| Publication | Experiment | Slope | *P* | Publication | Experiment | Slope | *P* |
| --- | --- | --- | --- | --- | --- | --- | --- |
| Albright_PO | S010_control_45 | 0.005 | 0.682 | Saghai_ASE | Bulk_Unplanted_42 | **0.020** | **<0.001** |
| Albright_PO | S018_control_45 | 0.002 | 0.880 | Saghai_ASE | Bulk_planted_63 | **0.013** | **0.008** |
| Calderon_ISME | Ulleraker_control_105 | 0.018 | 0.108 | Saghai_ASE | Bulk_Unplanted_63 | **0.017** | **<0.001** |
| Chen_EI | c_control_60 | **0.076** | **<0.001** | Saghai_ASE | Root_Unplanted_63 | **0.036** | **<0.001** |
| Costa_SBB | Rich_Mix_90 | **0.035** | **<0.001** | Saghai_ASE | Rhizosphere_Unplanted_63 | **0.045** | **<0.001** |
| Costa_SBB | Rich_Entero_90 | **0.037** | **<0.001** | Stadler_MB | c_METAGENOMIC_5 | **0.143** | **<0.001** |
| Costa_SBB | Poor_Mix_90 | 0.012 | 0.465 | Stadler_MB | c_TRANSCRIPTOMIC_5 | **0.112** | **<0.001** |
| Costa_SBB | Poor_Entero_90 | 0.022 | 0.265 | Tardy_EMR | c_control_42 | 0.017 | 0.124 |
| Costa_SBB | Poor_Burk_90 | 0.003 | 0.894 | Yan_ISME | soil_culture_56 | **0.059** | **<0.001** |
| Costa_SBB | Poor_Control_90 | 0.003 | 0.883 | Yan_ISME | rhizosphere_culture_56 | **0.059** | **<0.001** |
| Costa_SBB | Rich_Burk_90 | **0.021** | **0.010** | Yan_ISME | rhizosphere_culture_117 | **0.036** | **0.002** |
| Costa_SBB | Rich_Control_90 | **0.028** | **<0.001** | Yan_ISME | soil_culture_117 | **0.032** | **0.016** |
| Daval_MB | c_Control_49 | **0.030** | **<0.001** | Yan_ISME | suspension_suspension_1 | **0.044** | **<0.001** |
| Domeignoz_NC | c_HE_120 | **0.029** | **0.016** | Yu_ISME | c_control_7 | **0.141** | **<0.001** |
| Domeignoz_NC | c_LA_120 | **0.052** | **<0.001** | fluid_4_Zehavi_FiM | c_with_rumen | **0.107** | **<0.001** |
| Domeignoz_NC | c_HA_120 | **0.029** | **0.021** | Zegeye_AEM | c_aquatic_1 | **0.012** | **0.031** |
| Domeignoz_NC | c_LE_120 | 0.018 | 0.166 | Zegeye_AEM | c_aquatic_78 | **-0.015** | **<0.001** |
| Ernakovich_Biogeochemistry | Pasture_control_90 | **0.059** | **<0.001** | Zegeye_AEM | c_aquatic_106 | **0.057** | **<0.001** |
| Ernakovich_Biogeochemistry | Forest_control_90 | **0.028** | **0.004** | Zegeye_AEM | c_aquatic_15 | **0.068** | **<0.001** |
| Feng_ME | c_predisturbance_14 | **0.052** | **<0.001** | Zegeye_AEM | c_aquatic_22 | 0.017 | 0.097 |
| Feng_ME | c_postdisturbance_18 | **0.093** | **<0.001** | Zegeye_AEM | c_aquatic_36 | **0.033** | **<0.001** |
| Feng_ME | c_recovery_32 | **0.113** | **<0.001** | Zegeye_AEM | c_aquatic_50 | **0.048** | **<0.001** |
| Ferreira_ME | A_control_50 | -0.002 | 0.922 | Zegeye_AEM | c_aquatic_64 | -0.004 | 0.419 |
| Ferreira_ME | B_control_70 | -0.013 | 0.268 | Zegeye_AEM | c_soil_78 | **0.038** | **<0.001** |
| Ferreira_ME | B_control_50 | -0.004 | 0.787 | Zegeye_AEM | c_soil_106 | 0.009 | 0.057 |
| Ferreira_ME | A_control_70 | -0.007 | 0.591 | Zegeye_AEM | c_soil_15 | **0.070** | **<0.001** |
| Hol_Ecology | soilC_control_7 | 0.048 | 0.086 | Zegeye_AEM | c_soil_36 | **0.022** | **<0.001** |
| Hol_Ecology | soilD_control_7 | -0.016 | 0.544 | Zegeye_AEM | c_soil_50 | -0.002 | 0.597 |
| Hol_Ecology | soilB_control_7 | -0.014 | 0.605 | Zegeye_AEM | c_soil_64 | **0.047** | **<0.001** |
| Hol_Ecology | soilF_control_7 | 0.014 | 0.601 | Zha_PO | c_control_11 | -0.008 | 0.946 |
| Howard_FEMSML | c_control_21 | **0.077** | **<0.001** | Zhang_EST | c_control_10 | **0.129** | **<0.001** |
| Maron_AEM | c_control_42 | **0.026** | **0.028** | Zhang_PO | Sandy_control_84 | **0.020** | **0.025** |
| Saavedra_CJM | c_A_42 | -0.005 | 0.401 | Zhang_PO | Grassland_control_84 | **0.042** | **<0.001** |
| Saavedra_CJM | c_N_42 | **0.029** | **<0.001** | Zhang_SBB | c_control_30 | 0.014 | 0.347 |
| Saavedra_CJM | c_N_35 | **0.016** | **0.041** |  |  |  |  |
| Saavedra_CJM | c_N_28 | 0.012 | 0.088 |  |  |  |  |
| Saavedra_CJM | c_A_28 | -0.011 | 0.160 |  |  |  |  |

**Table S3** Heterogeneity test for meta-analysis

| Correlation | Mean Effect Size | df | Q_t_ | *P* |
| --- | --- | --- | --- | --- |
| Dilution Level - Richness | -1.2904 | 82 | 159.55 | <0.001 |
| Dilution Level - Shannon | -1.2374 | 82 | 150.54 | <0.001 |
| Dilution Level - MST | -1.1862 | 70 | 103.97 | 0.0042 |
| Richness- MST | 1.4721 | 69 | 133.07 | <0.001 |
| Shannon - MST | 1.2271 | 69 | 92.97 | 0.0288 |
| Mean rrn copy number - Dilution Level | 0.5616 | 70 | 122.82 | <0.001 |
| Mean rrn copy number - Richness | -0.6196 | 70 | 102.41 | 0.007 |
| Mean rrn copy number - Shannon | -0.5619 | 70 | 130.00 | <0.001 |
| Mean rrn copy number - MST | -0.5515 | 69 | 157.40 | <0.001 |

**Table S4** Introduction of moderators to explain the heterogeneity among different study in the meta-analysis

| Correlation | Moderator | Moderator | | | Residuals | | |
| --- | --- | --- | --- | --- | --- | --- | --- |
|  |  | df | Q_m_ | *P* | df | Q_e_ | *P* |
| Dilution Level - Richness | culture habitat | 4 | 3.7476 | 0.4412 | 78 | 155.8049 | <0.001 |
|  | regrowth day | 1 | 19.0473 | <0.001 | 81 | 131.4962 | <0.001 |
| Dilution Level - Shannon | culture habitat | 4 | 3.6473 | 0.4558 | 78 | 146.8962 | <0.001 |
|  | regrowth day | 1 | 19.0473 | <0.001 | 81 | 131.4962 | <0.001 |
| Dilution Level - MST | culture habitat | 4 | 18.3042 | 0.0004 | 66 | 114.7701 | <0.001 |
|  | regrowth day | 1 | 8.5268 | 0.0035 | 69 | 124.5474 | <0.001 |
| Richness- MST | culture habitat | 3 | 18.3042 | 0.0004 | 66 | 114.7701 | <0.001 |
|  | regrowth day | 1 | 8.5268 | 0.0035 | 68 | 124.5474 | <0.001 |
| Shannon - MST | culture habitat | 3 | 13.1999 | 0.0042 | 66 | 79.7749 | 0.1187 |
|  | regrowth day | 1 | 11.762 | 0.0006 | 68 | 81.2128 | 0.1307 |
| Mean rrn copy number - Dilution Level | culture habitat | 3 | 2.7838 | 0.4262 | 67 | 120.0362 | <0.001 |
|  | regrowth day | 1 | 3.713 | 0.054 | 69 | 119.1069 | <0.001 |
| Mean rrn copy number - Richness | culture habitat | 3 | 1.9078 | 0.5918 | 67 | 100.5004 | <0.001 |
|  | regrowth day | 1 | 6.4631 | 0.011 | 69 | 95.9452 | 0.0177 |
| Mean rrn copy number - Shannon | culture habitat | 3 | 3.6402 | 0.303 | 67 | 126.3577 | <0.001 |
|  | regrowth day | 1 | 4.4755 | 0.0344 | 69 | 125.5224 | <0.001 |
| Mean rrn copy number - MST | culture habitat | 3 | 0.8346 | 0.8412 | 67 | 156.5644 | <0.001 |
|  | regrowth day | 1 | 4.4851 | 0.0342 | 69 | 152.9138 | <0.001 |

**Table S5** The correlation between the relative abundance of abundant phyla/Classes and dilution level for soil culture habitat. The Pearson’s correlation is first calculated and transformed into effect size using Fisher’s z transformation. z_++_ is the estimate of mean effect size using meta regression The phylum *Proteobacteria* was separated into *Alphaproteobacteria*, *Betaproteobacteria*, *Gammaproteobacteria*, *Deltaproteobacteria* and unclassified *Proteobacteria*.

| Phylum/Class | z_++_ | sd | *P* |
| --- | --- | --- | --- |
| *Acidobacteria* | -0.866 | 0.0913 | <0.001 |
| *Actinobacteria* | -0.468 | 0.0913 | <0.001 |
| *Alphaproteobacteria* | -0.255 | 0.0913 | 0.005 |
| *Bacteroidetes* | 0.050 | 0.0941 | 0.593 |
| *Betaproteobacteria* | 0.357 | 0.0913 | <0.001 |
| *Chloroflexi* | -0.633 | 0.1018 | <0.001 |
| *Deltaproteobacteria* | -0.462 | 0.0913 | <0.001 |
| *Firmicutes* | -0.058 | 0.0937 | 0.533 |
| *Gammaproteobacteria* | -0.182 | 0.0913 | 0.046 |
| *Gemmatimonadetes* | -0.605 | 0.0955 | <0.001 |
| *Oligoflexia* | -0.255 | 0.1037 | 0.014 |
| *Planctomycetes* | -0.449 | 0.0913 | <0.001 |
| Unclassified *Bacteria* | -0.624 | 0.0913 | <0.001 |
| *Verrucomicrobia* | -0.178 | 0.0913 | 0.051 |

**Table S6** The ecosystem functions used and the reported BEF relationship in each DTE study. The classification of ecosystem functions was performed following the criteria as Roger et al. (2016)

| Study Number | Year | DOI | BEF | detail | Ecosystem Functions |
| --- | --- | --- | --- | --- | --- |
| 1 | 2001 | 10.1016/S0038-0717(01)00094-3 | complex | +/0/- | Degradation of Carbon Substrates; Nitrogen Cycling; Bacterial Activities; Stablity |
| 2 | 2004 | 10.1007/s00248-002-2043-7 | complex | +/0/- | Bacterial Activities; Nitrogen Cycling; Stablity |
| 3 | 2004 | 10.1016/j.soilbio.2004.03.001 | complex | +/0/- | Nitrogen Cycling |
| 4 | 2005 | 10.1007/s00248-004-0179-3 | positive |  | Invasion Resistance |
| 5 | 2006 | 10.1007/s00248-006-9105-1 | complex | +/0 | Degradation of Carbon Substrates; Plant Productivity Promotion; Invasion Resistance |
| 6 | 2006 | 10.1007/s00248-006-9033-0 | neutral |  | Degradation of Carbon Substrates |
| 7 | 2007 | 10.3354/ame047001 | neutral |  | Degradation of Carbon Substrates |
| 8 | 2010 | 10.1111/j.1461-0248.2009.01424.x | complex | 0/- | Plant Productivity Promotion; Nitrogen Cycling |
| 9 | 2010 | 10.1111/j.1462-2920.2007.01335.x | neutral |  | Nitrogen Cycling |
| 10 | 2011 | 10.1038/ismej.2010.119 | complex | +/0 | Bacterial Activities; Degradation of Carbon Substrates |
| 11 | 2012 | 10.1073/pnas.1109326109 | positive |  | Invasion Resistance |
| 12 | 2013 | 10.1007/s00374-013-0784-8 | complex | +/0 | Bacterial Activities; Degradation of Carbon Substrates |
| 13 | 2013 | 10.1007/s10533-012-9800-6 | complex | +/0/- | Degradation of Carbon Substrates |
| 14 | 2013 | 10.1038/ismej.2013.34 | complex | +/0 | Nitrogen Cycling |
| 15 | 2013 | 10.1111/1574-6941.12101 | complex | +/0 | Degradation of Carbon Substrates |
| 16 | 2013 | 10.1111/1758-2229.12053 | positive |  | Degradation of Carbon Substrates |
| 17 | 2013 | 10.1371/journal.pone.0076991 | positive |  | Invasion Resistance |
| 18 | 2014 | 10.1016/j.soilbio.2013.10.030 | complex | +/0 | Bacterial Activities; Degradation of Carbon Substrates |
| 19 | 2014 | 10.1111/1758-2229.12126 | complex | +/0 | Bacterial Activities; Stablity |
| 20 | 2014 | 10.3389/fmicb.2014.00230 | neutral |  | Degradation of Carbon Substrates |
| 21 | 2015 | 10.3389/fpls.2015.00485 | complex | +/0/- | Plant Productivity Promotion |
| 22 | 2015 | 10.1890/14-1001.1 | positive |  | Degradation of Carbon Substrates; Invasion Resistance |
| 23 | 2015 | 10.1890/14-2359.1 | positive | +/0 | Invasion Resistance |
| 24 | 2016 | 10.1016/j.soilbio.2016.04.024 | complex | +/0/- | Plant Productivity Promotion |
| 25 | 2016 | 10.1038/srep23012 | complex | +/- | Nitrogen Cycling; Degradation of Carbon Substrates |
| 26 | 2016 | 10.1002/ecy.1518 | neutral |  | Stablity; Degradation of Carbon Substrates; Nitrogen Cycling |
| 27 | 2016 | 10.1016/j.soilbio.2016.04.017 | positive |  | Stablity |
| 28 | 2016 | 10.1111/1365-2745.12585 | positive |  | Bacterial Activities; Degradation of Carbon Substrates |
| 29 | 2016 | 10.3389/fpls.2016.00759 | positive |  | Plant Productivity Promotion |
| 30 | 2017 | 10.1016/j.agee.2017.02.010 | complex | +/0 | Plant Productivity Promotion; Degradation of Carbon Substrates |
| 31 | 2017 | 10.1016/j.soilbio.2017.09.016 | complex | +/0 | Plant Productivity Promotion |
| 32 | 2017 | 10.5846/stxb201507211526 | complex | +/0/- | Plant Productivity Promotion; Nitrogen Cycling |
| 33 | 2017 | 10.1038/s41598-017-14803-0 | positive |  | Degradation of Carbon Substrates |
| 34 | 2017 | 10.13227/j.hjkx.201702058 | positive |  | Degradation of Carbon Substrates |
| 35 | 2018 | 10.1111/1751-7915.12870 | complex | +/0/- | Degradation of Carbon Substrates |
| 36 | 2018 | 10.1038/s41396-017-0003-y | positive |  | Invasion Resistance; Degradation of Carbon Substrates |
| 37 | 2018 | 10.1080/1065657X.2018.1438933 | positive |  | Plant Productivity Promotion |
| 38 | 2018 | 10.1111/mec.14895 | positive |  | Bacterial Activities |
| 39 | 2018 | 10.1128/AEM.02738-17 | positive |  | Bacterial Activities |
| 40 | 2019 | 10.1016/j.soilbio.2019.05.008 | complex | +/- | Nitrogen Cycling; Degradation of Carbon Substrates |
| 41 | 2019 | 10.1038/s41396-019-0356-5 | complex | +/0 | Bacterial Activities |
| 42 | 2019 | 10.1039/c9em00327d | negative |  | Degradation of Carbon Substrates |
| 43 | 2019 | 10.1007/s11356-019-05781-z | positive |  | Degradation of Carbon Substrates |
| 44 | 2019 | 10.1016/j.soilbio.2018.10.013 | positive |  | Invasion Resistance |
| 45 | 2019 | 10.1264/jsme2.ME18138 | positive | +/0 | Plant Productivity Promotion |
| 46 | 2019 | 10.13671/j.hjkxxb.2018.0013 | positive |  | Degradation of Carbon Substrates |
| 47 | 2020 | 10.1007/s00248-020-01502-z | complex | +/0/- | Bacterial Activities; Invasion Resistance |
| 48 | 2020 | 10.1016/j.soilbio.2020.107893 | complex | +/0/- | Plant Productivity Promotion |
| 49 | 2020 | 10.1038/s41467-020-17502-z | complex | +/0/- | Bacterial Activities; Invasion Resistance |
| 50 | 2020 | 10.1038/s41559-019-1084-y | complex | +/0 | Bacterial Activities; Nitrogen Cycling; Other elementary cycling |
| 51 | 2020 | 10.1111/1751-7915.13634 | complex | +/0 | Plant Productivity Promotion |
| 52 | 2020 | 10.1111/pce.13712 | complex | +/0 | plant production |
| 53 | 2020 | 10.1139/cjm-2019-0412 | complex | +/0 | Degradation of Carbon Substrates; Bacterial Activities |
| 54 | 2020 | 10.1371/journal.pone.0224641 | complex | +/0 | Bacterial Activities |
| 55 | 2020 | 10.1016/j.envint.2020.105766 | positive |  | Plant Productivity Promotion |
| 56 | 2020 | 10.1038/s41598-020-63091-8 | positive |  | Bacterial Activities |
| 57 | 2021 | 10.1007/s10533-020-00736-w | complex | +/0 | Degradation of Carbon Substrates |
| 58 | 2021 | 10.1111/ele.13769 | complex | +/0 | Bacterial Activities; Plant Productivity Promotion |
| 59 | 2021 | 10.1111/nph.17065 | complex | +/0/- | Plant Productivity Promotion |
| 60 | 2021 | 10.1016/j.jhazmat.2020.124385 | negative |  | Degradation of Carbon Substrates |
| 61 | 2021 | 10.3390/su13041685 | neutral |  | Nitrogen Cycling |
| 62 | 2021 | 10.1016/j.envpol.2020.116374 | positive |  | Degradation of Carbon Substrates |
| 63 | 2021 | 10.5846/stxb201811222534 | positive |  | Bacterial Activities; Degradation of Carbon Substrates |
| 64 | 2022 | 10.1016/j.scitotenv.2022.153082 | positive |  | Nitrogen Cycling; Degradation of Carbon Substrates |
| 65 | 2022 | 10.1016/j.scitotenv.2022.154239 | positive |  | Degradation of Carbon Substrates |
| 66 | 2012 | 10.1016/j.pedobi.2012.06.001 | neutral |  | Bacterial Activities |
